# Detecting child sexual abuse in child and adolescent psychiatry: a survey study of healthcare professionals’ assessment practice

**DOI:** 10.1186/s13033-024-00632-y

**Published:** 2024-04-18

**Authors:** Margrethe Seeger Halvorsen, Signe Hjelen Stige, Jorunn E. Halvorsen, Per-Einar Binder, Elida Måkestad, Ane Ugland Albaek, Ann Christin Andersen

**Affiliations:** 1 Department of Psychology, University of Oslo, Oslo, Norway; 2 Department of Clinical Psychology, University of Bergen, Bergen, Norway; 3 Øyane DPS, Bergen Hospital Trust, Bergen, Norway; 4 Bergen Hospital Trust, Bergen, Norway; 5 Faculty of Health and Sport Sciences, Department of Psychosocial Health, University of Agder, Kristiansand, Norway; 6 Regional Centre for Child and Youth Mental Health and Child Welfare (RKBU), Department of Mental Health, Faculty of Medicine and Health Sciences, Norwegian University of Science and Technology (NTNU), Trondheim, Norway; 7 Department of Child and Adolescent Psychiatry, M?re og Romsdal Hospital Trust, Volda Hospital, Volda, Norway

**Keywords:** Child sexual abuse (CSA), Healthcare professionals, Child and adolescent psychiatry (CAP), Uncover, Detect, Facilitate

## Abstract

**Background:**

Research shows that only around half of all survivors of child sexual abuse (CSA) disclose the abuse during childhood and adolescence. This is worrying, as CSA is related to substantial suffering later in life. The proportion of children and adolescents who have been exposed to CSA is significantly higher in Child and Adolescent Psychiatry (CAP) than in the general population. Healthcare professionals report that uncovering CSA is a complex and challenging task. However, we know little about how they proceed when uncovering CSA. More knowledge of healthcare personnel’s experience is therefore necessary to facilitate and increase CSA disclosure. The study aims to explore how CAP healthcare professionals in Norway proceed when assessing and detecting CSA, how they experience this work, and what hinders or facilitates their efforts.

**Methods:**

The study employed a mixed method approach. Data was collected through an anonymous online survey, generating both quantitative and qualitative data. The sample consisted of 111 healthcare professionals in CAP, of whom 84% were women, with a mean age of 40.7 years (range 24–72; sd = 10.8). Mean years of CAP clinical experience were 8.3 years (range 0–41; sd = 7.5). The quantitative data was analysed using descriptive statistics, correlations, and independent sample t-tests, while the qualitative data was analysed using a team-based qualitative content analysis.

**Results:**

The results showed that detection of CSA was viewed as an important, but complex task in CAP, and the existing procedures were deemed to be insufficient. The therapists mostly felt confident about how to proceed when they suspected or detected CSA, yet they seldom detected CSA. In their initial assessment they applied standardised procedures, but if their suspicion of possible CSA persisted, they seemed to rely more on clinical judgement. Specific challenges and facilitators for CSA detection were identified, both in the individual and in the organisation.

**Conclusions:**

The study highlights the challenges and complexities healthcare professionals and the CAP system face when assessing CSA, which may account for the low detection rate. The results show that healthcare professionals believe room for clinical autonomy and targeted competence development may improve CSA detection. Additionally, the findings suggest a need for CAP to define roles and responsibilities within and between agencies.

**Supplementary Information:**

The online version contains supplementary material available at 10.1186/s13033-024-00632-y.

## Introduction

International reviews and meta-analyses indicate that as many as 18–20% of females and 8–10% of males are exposed to sexual abuse in childhood (CSA; Alaggia et al. 2019; Pereda et al. 2009). Yet only around half of the survivors disclose the abuse during childhood or adolescence (McElvaney 2015), and many survivors never tell. This non-disclosure rate is alarming considering the significant negative health implications of CSA, such as somatic and mental illnesses, risk behaviour, and premature death (Briere and Elliott 2003; Coles et al. 2015; Felitti et al. 1998; Fitzgerald 2022; Hughes et al. 2017; Jonas et al. 2011; Targum and Nemeroff 2019; Trickett et al. 2011). Research also shows that early disclosure and a constructive response to disclosure may reduce the risk of mental illness, while delayed disclosure and non-disclosure are associated with an increased risk of mental health problems (Easton 2019; Hébert et al. 2009; Kogan 2004; Swingle et al. 2016). Interventions deemed to increase the detection rate are thus of major importance.

Research and clinical practice reveal greater prevalence of CSA among children in mental healthcare services than in the general population (Robin et al. 2023; Spataro et al. 2004), yet health professionals are rarely the first to learn about the abuse (Brattfjell and Flåm 2019; Kogan 2004; Lahtinen et al. 2018; Manay and Collin-Vézina 2021). Abuse is often unknown and only rarely a cause of referral or primary contact. Research and clinical experience shows that abused children and adolescents more commonly present with other problems, such as behavioural problems, avoidant behaviour, substance misuse, self-harm or eating disturbances (Keeshina et al. 2014), making CSA detection more difficult.

For several years, new strategies to enhance CSA detection have been implemented in mental healthcare services globally and in Norway. Healthcare professionals in Norwegian Child and Adolescent Psychiatry (CAP) are required to assess traumatic experiences, including sexual abuse, as part of their basic assessment procedures. In addition, they must adhere to laws mandating reporting suspected maltreatment, such as CSA, to relevant authorities (i.e., law enforcement (LE) and Child Protective Services (CPS) (Lovdata/Legal Information 2020). Norwegian healthcare personnel are also bound by confidentiality laws. Differentiating between these two types of concern while evaluating complex cases may be challenging (Ohnstad and Gudheim 2019; Meysen & Kelly, 2018).

A previously published meta-synthesis shed light on the highly demanding and complex processes involved in CSA detection for both survivors and healthcare professionals (Stige et al. 2022). Strategies to aid professionals in this daunting task are vital; however, we lack adequate knowledge about their experiences and challenges in detecting CSA. This underscores the need for increased knowledge to improve CSA detection in the context of CAP. This would also represent a key secondary preventive strategy (Easton 2019).

### Aim of the present study

The present study’s aim is twofold: firstly, to increase knowledge of how CAP healthcare professionals in Norway proceed in assessing and detecting CSA in their clinical work; and secondly, to explore how they experience this work and what hinders or facilitates their efforts.

## Method

### Design and data collection

For the present study, we used an anonymous survey to generate both quantitative and qualitative data. The purpose of the survey was to shed light on the decision-making process leading to the assessment of CSA within CAP, the assessment procedures used, and what the therapists found most useful and detrimental in this process. The survey was developed for this study and consisted of 10 questions mapping demographics and professional background, and 18 questions mapping assessment practice and experience of assessing CSA. The questions were developed on the basis of previous research (Albaek et al. 2018; Gawel et al. 2015; Nouman et al. 2020; Stige et al. 2022) and our own clinical experience. Ratings were given on a scale of 1–5 (low-high/rarely-frequently). In addition, 16 open-ended questions were included, to give the therapists an opportunity to elaborate on their assessment practice and experience from assessing CSA. The survey was piloted among experienced therapists associated with the University of Bergen, before being distributed. This led to an adjustment of the wording of some of the questions. For the full survey, see Supplementary material 1. SurveyXact was used to distribute and manage the survey.

### Recruitment procedure and sample

The survey was distributed through several channels: Information about the survey and a link to SurveyXact were posted on two Norwegian Facebook groups for psychologists. In addition, the heads of several CAP units in Norway, representing all four health regions, distributed information about the survey to their therapists. Due to the recruitment procedure and resulting convenience sample, the exact number of potential respondents is unknown. Considering the total number of CAP therapists in Norway, the response rate was low. Moreover, it is possible that those who completed the survey were therapists with a special interest or engagement in the topic of child sexual abuse. Limitations relating to this will be discussed.

The sample consists of 111 respondents, of whom 84% were women (i.e., all participants identified as binary), with a mean age of 40.7 years (range 24–72; sd = 10.8). Mean years of CAP clinical experience were 8.3 years (range 0–41; sd = 7.5). Most of the sample group were psychologists (See Table 1). 62% of the sample group were specialists within their field. All health service regions in Norway were represented, with 34.2% of the respondents working in Health Region West, 27.9% in Health Region South-East, 23.4% in Health Region Mid-Norway, and 14.4% in Health Region North.

**Table 1:** Professional background (*N* = 111)

Professions	N	%
Psychologist	80	72.1
Medical doctor	17	15.3
Social worker	5	4.5
Pedagogue	3	2.7
Other	6	5.4

### Data analysis

To gain an overview of the dataset, descriptive statistics was run for the quantitative data using SPSS (version 29). A Pearson correlation coefficient was computed for all continuous variables (i.e., age, years of experience, weekly consultations, assessment practice, and experience of assessing and uncovering CSA). Independent sample t-tests were performed to evaluate whether there was a difference in assessment and detecting practices between (a) female and male therapists and (b) between therapists being a specialist or not. Subsequently, the responses to the open-ended questions were analyzed using content analysis (Graneheim and Lundman 2004) and team-based qualitative data analysis (Binder et al. 2012). The initial step involved the first and second author familiarizing themselves with the qualitative data, subsequently coding all responses to each open-ended question and grouping them systematically. This analysis was then presented to the rest of the research team, together with descriptive statistics. The combined analyses was then discussed and synthesized in a sequence of team meetings and shared documents in Teams. Thus, our findings were derived from the descriptive statistics, the correlation analysis, and the qualitative content analysis, as presented below.

### Trustworthiness

The author team consists of one male and six female, Caucasian, Norwegian-speaking members. All seven authors are healthcare professionals, and together they have extensive clinical experience in child, adolescent, and adult mental health services. They share an engagement in research and treatment of psychological trauma and sexual abuse, thus securing sufficient access to the phenomena under study (i.e., ‘Engagement’; Stige et al. 2009). This common engagement was the background for establishing this research team – but also called for careful reflexive processes to ensure our engagement did not interfere with the rigor of the research process. Five authors are clinical psychologists, one is an organisational psychologist, and one is a child and adolescent psychiatrist. Five team members also have academic positions, of whom two are professors in clinical psychology, two are associate professors in organisational and clinical psychology, respectively, and one is assistant professor in medicine and health sciences. This variance supported reflexive processes and was used actively throughout the research process – e.g., by discussing expectations based on clinical experience in light of existing research literature.

### Ethics

The project was approved by the Norwegian Agency for Shared Services in Education and Research (Sikt, reference 251846). Questions were formulated to ensure that participants could answer without the risk of later identification or violation of patient and therapist confidentiality.

## Findings

The findings are presented and organised within three topics: (1) the organisational context in which the assessment of CSA takes place; (2) the therapists’ descriptions of how they proceed to assess and detect CSA; and (3) how they evaluate the adequacy of their practice to detect CSA. The results are presented in accordance with the questionnaire and quantitative data, with supplementary excerpts from the open answers. Where we present quantitative findings, the statements are taken from the questionnaire. All quotes are taken from the participants’ statements in the qualitative data material. For correlations (i.e., age, years of experience, weekly consultations, assessment practice, and experience of assessing and uncovering CSA) see Table 2.

**Table 2:** Numbers, Means, Standard deviations and Pearson correlations for continuous variables

Variable	N	M	SD	1.	2.	3.	4.	5.	6.	7.	8.	9.	10.	11.	12.	13.	14.
1. Age	111	40.69	10.83	-													
2. Year graduated	110	2007	10.57	**− 0.97** ^**^	-												
3. Years in CAP	111	8.33	7.49	.82**	− 0.80**												
4. Percent position in CAP	111	91.53	20.80	− 0.13	0.16	− 0.11	-										
5. Number of consultations/ week	111	13.23	5.00	− 0.14	0.15	− 0.01	0.58*	-									
6. Frequency assessment CSA	109	2.52	0.95	− 0.22*	0.20*	− 0.18	0.16	.23*	-								
7. Know what to do when suspect CSA	101	3.77	0.84	0.11	− 0.16	0.14	0.02	0.07	0.08	-							
8. Frequency new assessment CSA	103	2.33	0.80	0.05	− 0.03	0.07	0.18	.25*	0.43**	0.07	-						
9. Frequency uncovering CSA	89	1.49	0.64	− 0.11	0.11	− 0.09	− 0.08	0.13	0.40**	0.20	0.41**	-					
10. Know what to do when uncover CSA	90	3.71	0.82	0.23**	− 0.24*	0.22*	− 0.09	− 0.05	0.01	0.68**	0.17	.28**	-				
11. Satisfied current practice (CSA)	89	3.92	0.92	− 0.09	0.08	− 0.06	− 0.11	0.03	0.27*	− 0.05	.29**	0.34**	0.06	-			
12. Support workplace	89	4.02	0.75	0.23*	− 0.22*	0.13	− 0.03	0.01	0.00	.23*	− 0.03	0.16	0.27*	0.43**	-		
13. CSA is topic at workplace	88	2.90	1.14	− 0.02	0.02	− 0.08	− 0.09	− 0.04	0.17	0.10	0.33**	0.38**	0.26*	0.44**	0.29**	-	
14. CAP’s role in CSA uncovering	87	4.24	1.00	− 0.13	0.11	− 0.08	− 0.11	− 0.09	0.03	0.03	− 0.09	0.18	0.03	0.27*	0.04	0.13	-

*Note* *p < .05. **p < .01 (2-tailed)

### Organisational context – detection of CSA is a crucial task in CAP, but difficult to achieve

This first topic encompasses the organisational context in which potential detection of CSA takes place. Detection of CSA was viewed as an essential task in CAP, and assessment of CSA was part of the initial, standardised procedures, the therapists performed regularly. Generally, the therapists seemed to be confident about how to proceed when they suspected or detected CSA, but despite their efforts, they seldom detected CSA. And there was a huge variation in how CSA was addressed in their organisations.

Most therapists (84%) believed that CAP plays an important role in detecting CSA. Yet their open-ended answers to the same question revealed a more nuanced picture: Some therapists believed that CAP played a vital role in the detection of CSA, while others expressed how detection of CSA was not a responsibility they could assume, or that other agencies were better equipped to detect CSA:

I think those who see the children every day, in school, kindergartens, and child protective services have the most important role in the detection of sexual abuse. If abuse has not been detected before the child comes to the CAP clinic, then this will be our task.

When asked what initiated an assessment of CSA, 89% of the participants indicated that this was part of the procedure for basic assessment within the first two sessions. In line with this, 85% of the participants reported assessing for CSA at least once a month, ranging from several times a week (18%), to every week (29%), and to every month (38%). How often the therapists assessed for CSA correlated with age (*r* = − .22, *p* < .05 i.e., younger therapists assessed more often), time since graduation (*r* = .20, *p* < .05; those with shorter time since graduation assessed more often), and number of consultations per week (*r* = .58, *p* < .05; those with higher number of consultations per week assessed more often). Female therapists (M = 2.61, sd = 0.91) assessed for CSA significantly more often than male therapists (M = 2.06, sd = 1.03, t(107) = -2.24, *p* = .027. Comparison between genders on the dependent variables is presented in Table 3.

**Table 3:** T-test independent sample. Gender differences on all continuous variables

	**Male**			**Female**						
**Variables**	***N***	***M***	***SD***	***N***	***M***	***SD***	***df***	***t***	***p***	**Cohen’s** ***d***
1. Age	18	45.11	11.79	93	39.84	10.49	109	1.19	0.058	0.49
2. Year graduated	18	2004	11.50	92	2008	10.31	108	-1.51	0.135	0.39
3. Years in CAP	18	11.28	10.80	93	7.76	6.60	109	1.85	0.068	0.48
4. Percent position in CAP	18	89.17	28.40	93	91.99	19.16	109	-0.53	0.600	0.14
5. Number of consultations/ week	18	11.78	6.48	93	13.51	4.65	109	-1.35	0.181	0.35
6. Frequency assessment CSA	17	2.06	1.03	92	2.61	0.91	107	-2.24	**0.027**	0.59
7. Know what to do when suspect CSA	17	3.59	0.87	84	3.81	0.83	99	-1.00	0.322	0.27
8. Frequency new assessment	15	2.00	0.76	88	2.39	0.79	101	-1.75	0.083	0.49
9. Frequency uncovering CSA	14	1.29	0.47	75	1.53	0.66	87	-1.33	0.187	0.39
10. Know what to do when uncover CSA	15	3.53	0.83	75	3.75	0.82	88	-0.91	0.307	0.26
11. Satisfied current practice (CSA)	15	3.93	1.10	74	3.92	0.89	87	0.06	0.956	0.02
12. Support workplace	15	4.13	1.06	74	4.00	0.68	87	0.62	0.535	0.18
13. CSA is topic at workplace	15	3.27	0.96	73	2.82	1.16	86	1.39	0.168	0.39
14. CAP’s role in CSA uncovering	15	4.00	1.20	72	4.29	0.96	85	-1.03	0.307	0.29

In line with the detection of CSA being considered a significant task for therapists in CAP, 61% reported CSA as a topic of discussion at the workplace at least once a month. Some therapists reported that these discussions were part of routine practice, while others reported that they were initiated when relevant. There was significant variation, however, with 11% of the therapists reporting that CSA was on the agenda less than once a year. The topic was discussed most often in relation to specific cases, or procedures for assessment, and less often in conjunction with discussion of their practice:

Every week it is confirmed that we should do basic assessment, including an assessment of child sexual abuse; “Everyone is asked”. But beyond that this is totally self-evident, there are rarely discussions regarding whether we should ask everyone, or if we should do something different in assessing/detecting sexual abuse, as I perceive it.

Many therapists reported mostly knowing what to do when CSA was suspected (68%) and detected (63%). The rest reported being uncertain about how to proceed. When elaborating on this uncertainty, the therapists mentioned the role of CAP in reporting CSA to the police, how to appraise the risk of repetition and the urgency of reporting, whether and when to inform caregivers, and how to establish collaboration with the child/adolescent and parents concerning the reporting of abuse. Knowing what to do when CSA was detected correlated positively with age (*r* = .23, *p* < .01), time since graduation (*r* = .24, *p* < .05), length of time working in CAP (*r* = .22, *p* < .05), knowing what to do when suspecting CSA (*r* = .68, *p* < .01), how often they detected CSA (*r* = .28, *p* < .01), perceived support in the workplace (*r* = .27, *p* < .05), and how often CSA was a topic of discussion at the workplace (*r* = .26, *p* < .05). Furthermore, therapists who were specialists (M = 3.94, sd = 0.78) were more likely to know what to do when they suspected CSA than therapists who were not specialists (M = 3.50, sd = 0.86, t(99) = -2.62, *p* = .010), as well as when they uncovered CSA (specialists; M = 3.89, sd = 0.75 vs. non-specialists; M = 3.39, sd = 0.86, t(88) = -2.89, *p* = .005). Comparison between therapists being specialists or not on the dependent variables is presented in Table 4.

**Table 4:** T-test independent sample. Differences on all continuous variables between therapists being a specialist or not

	**Not being a specialist**			**Being a specialist**						
**Variables**	***N***	***M***	***SD***	***N***	***M***	***SD***	***df***	*t*	***p***	**Cohen’s** ***d***
1. Age	42	33.14	8.39	69	45.29	9.53	109	-6.81	**< 0.001**	1.33
2. Year graduated	41	2014	8.78	69	2003	9.34	108	6.16	**< 0.001**	1.21
3. Years in CAP	42	3.62	4.71	69	11.20	7.44	109	-5.92	**< 0.001**	1.16
4. Percent position in CAP	42	94.29	19.40	69	89.86	21.57	109	1.09	0.278	0.21
5. Number of consultations/ week	42	13.29	4.70	69	13.19	5.21	109	1.00	0.921	0.02
6. Frequency assessment CSA	42	2.63	0.84	69	2.46	1.01	107	0.85	0.395	0.17
7. Know what to do when suspect CSA	38	3.50	0.86	63	3.94	0.78	99	-2.62	**0.010**	0.54
8. Frequency new assessment CSA	39	2.38	0.71	64	2.30	0.85	101	0.54	0.590	0.11
9. Frequency uncovering CSA	34	1.47	0.61	55	1.51	0.66	87	− 0.27	0.785	0.06
10. Know what to do when uncover CSA	33	3.39	0.86	57	3.89	0.75	88	-2.89	**0.005**	0.63
11. Satisfied current practice (CSA)	33	3.85	0.87	56	3.96	0.95	87	− 0.57	0.569	0.13
12. Support workplace	33	3.85	0.67	56	4.13	0.79	87	-1.69	0.095	0.37
13. CSA is topic at workplace	32	2.91	1.03	56	2.89	1.20	86	0.05	0.958	0.01
14. CAP’s role in CSA uncovering	33	4.30	0.92	54	4.20	1.05	85	0.45	0.656	0.10

Even though they frequently assessed CSA and mostly knew how to proceed, therapists relatively seldom reported uncovering CSA. Only 8% detected CSA on a monthly basis, and the rest reported detecting CSA only a couple of times a year, or less. The frequency with which therapists uncovered CSA correlated positively with how often they assessed for CSA (*r* = .40, *p* < .01), how often they reassessed for CSA (*r* = .41, *p* < .01), how often CSA was a topic of discussion at the workplace (*r* = .38, *p* < .01), knowing what to do when CSA was detected (*r* = .28, *p* < .01), and how satisfied they were with workplace procedures regarding assessment of CSA (*r* = .34, *p* < .01). Age, gender, work experience and being a specialist or not was not related to the frequency of CSA detection.

### Facing complexity – from following standardised procedures to relying on clinical judgement

The second topic encompasses the therapists’ description of how they proceeded to detect CSA. They seemed to use a variety of approaches in their assessment, detection and reporting of CSA. Generally, a set of standardised procedures seemed to be available for initial assessment and reporting to legal instances, but therapists seemed to rely more on their clinical judgement in their ongoing assessment. Detection of CSA had a variety of consequences for treatment.

Most therapists indicated using both standardised measures (91%) and open conversation with the child (88%), when assessing for CSA. Some (24%) indicated using unstructured assessments such as playing, drawings, etc. Answers to the open questions confirmed that the therapists relied on several sources of information when assessing CSA, adjusting the assessment process to the individual client. Many therapists thus expressed that they found it difficult to pinpoint the most important assessment method.

The process following initial assessment of CSA was more unstructured and depended on the individual therapists’ clinical judgement. Only one third of the therapists indicated performing a repeated assessment of CSA at least once a month. The remaining therapists indicated doing this more rarely. In the basic assessment, the therapists indicated the use of CATS (NKVTS, 2016; Sachser et al. 2017). When pursuing suspected CSA and repeating assessments, therapists preferred direct conversation with the child/adolescent.

Standardised assessment does not necessarily give us an answer as to whether child sexual abuse has occurred (but it might often do!), and I believe that as a clinician one has to ask the right questions in the “right” context. This requires clinical judgement and “experience” in addressing this as a topic in treatment.

The therapists seemed to be continuously attentive to the possibility that new information or changes in symptoms and/or functional impairment might elicit a need for more assessment (this includes an increase or change in symptom load, sudden or unexpected deterioration, or uncertainty about information missed in the primary assessment). When asked what was most decisive for initiating this second assessment, therapists reported that suspicion of CSA was often maintained or elicited through a combination of several information sources, including an experience of the young person not managing or wanting to be honest: “Everything that elicits suspicion of sexual abuse: utterances from the client, information from others, symptoms, input from other therapists, etc.” When forced to choose what was most decisive for initiating a second assessment, therapists indicated client utterances (41%), the emergence of new information (28%), clinical intuition (14%) and clinical observation (11%).

In cases where CSA was detected, 78% of therapists reported to the CPS, and 37% were actively involved in reporting the event to the police. When asked what consequences detection of CSA had for treatment, 26% of therapists reported pausing treatment while sorting out the roles of the agencies involved, 33% continued treatment without focusing on the trauma, and 48% reported adjusting the treatment focus to include trauma symptoms and trauma history. A few therapists mentioned in the open answers that they sometimes did not report to the CPS or the LE when working with adolescents in cases where the abuse had ended, and the adolescent did not want these authorities to be involved. Some mentioned they tried to involve the adolescents’ caregivers: “Often, adolescents do not want caregivers to be informed. We, therefore, work actively to get permission to inform the caregivers so that both the caregivers and the adolescents can get a better understanding of (the adolescent’s) difficulties.”

### “It takes a lot for a child to tell” – existing procedures are not sufficient to manage the challenge

The third topic encompasses how the therapists evaluated the adequacy of their procedures to detect CSA. Overall, the therapists experienced sufficient support to do this work, but they felt the task of detecting CSA was complex, and that the existing procedures were inadequate. Time pressure and fear of making mistakes were two of several barriers to detection. Having enough time to build a safe relationship, more competence in talking to children/adolescents about CSA, and training in trauma treatment were potential facilitative factors.

The majority of the therapists (80%) reported mostly having the support needed for assessing CSA. Perceptions of support in the workplace correlated positively with age (*r* = .23, *p* < .05), time since graduation (*r* = .22, *p* < .05; i.e. older therapists and therapists with longer experience felt they had more support), knowing what to do when CSA was suspected (*r* = .23, *p* < .05), knowing what to do when CSA was detected (*r* = .27, *p* < .05), how often CSA was a topic of discussion at the workplace (*r* = .29, *p* < .01), and how satisfied they were with workplace procedures regarding assessment of CSA (*r* = .43, *p* < .01). The qualitative data showed that the therapists perceived support to be related mostly to the experience of not being alone with the responsibility for the child and with the decisions on how to proceed, i.e., knowing that there was trauma expertise available at the workplace, that colleagues and management were available when needed, supervision, being part of a team in which one could discuss cases, and external instances available that could provide information and recommendations. However, only 25% reported that they believed existing assessment practices contributed to the detection of CSA to a great extent. Satisfaction with current assessment practices correlated positively with how often they assessed for CSA (*r* = .27, *p* < .05), how often they reassessed for CSA (*r* = .29, *p* < .01), how often they uncovered CSA (*r* = .34, *p* < .01), how often CSA was a topic of discussion at the workplace (*r* = .44, *p* < .01), how much support they experienced in the workplace (*r* = .43, *p* < .01), and the extent to which they recognised CAP as having an important role in the uncovering process (*r* = .27, *p* < .05). Therapists pointed to the complexity of the task at hand, and the importance of not overrating therapists’ ability to detect CSA within the given CAP framework:

It is difficult to assess, not necessarily because the tools are bad (or at least not only because of that), but also because it takes a lot for a child to tell, almost regardless of how we proceed. This is not acknowledged sufficiently, and I believe there is an excessive belief that therapists in child and adolescent psychiatry are good at detecting sexual abuse. Clarification of expectations and supervision of parents (and other agencies) is a large part of my job in these cases.

Time constraints were reported as the most important barrier to detection of CSA (45%). The therapists felt time limitations made it difficult to establish the safe therapeutic relationship considered necessary for the child to be able to talk about CSA. One therapist wrote: “Unfortunately, we try, and we ask, and we want to be a safe place to tell, but we become less relevant because we don’t have time to establish a secure relationship.” Other important barriers were lack of knowledge (14%), fear of making mistakes (8%), and fear of wrongfully suspecting CSA (6%). In addition, in the open answers, therapists reported that the fear of creating false memories and the fear of making it worse for the child were important barriers to detection:

It is difficult to know how to collect information about sexual abuse. I have experienced doing CATS without any affirmative answers, while having strong indications for abuse through drawings/information from caregivers/observation. It is difficult to progress in such cases, and it is difficult to find a balance where you collect information without being too asking/leading in your questions. It is challenging to continue working in cases where the suspicion is strong. I experience that the children withdraw if you become too eager to figure out whether something might have happened.

Focusing on cases where therapists detected CSA, the majority (60%) experienced direct conversation with the child as most helpful, while a third found standardised measures most helpful. When invited to use their experience to suggest what could contribute to increasing detection rates in CAP, a third of the therapists believed clearer procedures following the initial assessment could facilitate increased detection of CSA:

It is helpful that everyone *HAS* to use CATS. BUT, when this is done as part of the initial assessment, you most often do not have a sufficiently strong therapeutic alliance, which results in the children/adolescents not feeling sufficiently safe to tell about abuse. Therefore, there should perhaps be a reminder of a new conversation with this focus at the 12-week evaluation, for example.

It is important to note, however, that some therapists cautioned that procedures, if not used with a high degree of clinical expertise, might lead therapists to drop suspicion of CSA too early in cases where CATS failed to indicate trauma exposure.

I am a bit worried that when it comes down to it, therapists will put the topic/the possibility that sexual abuse has occurred away if someone denies exposure during the assessment. That we are only searching for other diagnostic explanations (than sexual abuse) for the symptoms/level of functioning we observe.

However, the most significant facilitator to increase detection of CSA was training in how to talk to children and adolescents about CSA (71%). Therapists also reported more flexibility and less time pressure (56%), increased knowledge of CSA and detection (55%), as well as increased support from colleagues (21%) and management (13%), as important facilitators for the detection of CSA:

I experience that there is a lot of knowledge about sexual abuse in child and adolescent psychiatry; the procedures are there, strictly speaking, but working with traumatic experiences requires more than procedures – there could be a lot of reasons why children/adolescents don’t tell about abuse even if we assess this, and then we need valid knowledge about trauma in order to initiate a dialogue about this and to be secure about how to ask about these types of experiences.

Improved cooperation with other agencies (52%), including knowing that the police prioritised investigating abuse (31%), and securing interdisciplinary forums where suspicion could be discussed (44%), were also mentioned as potential facilitators for the detection of CSA.

## Discussion

In the present study we have explored how healthcare professionals in CAP in Norway proceed in assessing and detecting CSA in their clinical work, and how they experience this endeavour. The results showed that detection of CSA was viewed as an important task in CAP, but that it was difficult to achieve. The therapists mostly seemed to be confident about how to proceed when they suspected or detected CSA. Yet they seldom detected CSA. In their initial assessment, they applied a set of standardised procedures, but if their suspicion of possible CSA persisted in their subsequent work with a child/adolescent, they seemed to rely more on clinical judgement and preferred direct conversation with the young person. Overall, most therapists experienced having support in their work, but the task of detecting CSA seemed complex, and the existing procedures were not deemed to be sufficient.

The results highlight the complexity of uncovering CSA and emphasise some of the challenges healthcare professionals and CAP institutions must resolve, to enable the young person to disclose CSA experiences. The results align with and expand existing knowledge of how healthcare professionals conduct this demanding clinical task and what might facilitate this work (Stige et al. 2022). The current data is not sufficient to provide conclusive answers, but our results do reveal some important dilemmas and potential areas of development to increase CSA detection rates in CAP. In the following, we will discuss some of the challenges and dilemmas healthcare professionals and management face when balancing at the intersection between standardised procedures and clinical judgement.

### The individual healthcare professional: how and when should I allow my clinical judgement to override standardised procedures?

Overall, our findings indicated an inherent tension that healthcare professionals experienced between the use of clinical judgement and standardised procedures – between their loyalty towards their clients and their adherence to workplace regulations. In line with this, the results showed that healthcare professionals emphasised sufficient time to establish a strong alliance with the child/adolescent, and allowance to use their clinical judgement to adjust the assessment and treatment to each child. At the same time, they asked for more procedures and additional training in how to talk to young people about CSA as a means to facilitate CSA detection. This illustrates how demanding these cases may be for healthcare professionals – and the significance of paying attention to the nuances and complexity of the processes leading to CSA detection.

Clinical judgement can take the form of phronesis – a pragmatic, context-dependent and action-oriented form of knowing (Kinsella and Pitman 2012), that hinges on experience and contextual understanding within a field. Pitman and Kinsella (2012) point out that the need for phronesis becomes particularly apparent when the economic and technological context surrounding professional occupations results in a shift from an emphasis on the professional’s *responsibility* in practice towards *accountability* for one’s practice. While accountability towards organizational standards and legal regulations is essential on a group level to safeguard patient rights and prevent arbitrariness, professionals, at an individual level, must exercise responsible judgement considering the patient’s unique life context.

In line with this, time pressure was reported as the most important barrier to CSA detection, while 60% experienced conversation with the young person to be most helpful in determining suspected CSA. In accordance with results in previous research (e.g., Stige et al. 2022), over half of the participants believed flexibility and time to establish a strong therapeutic relationship facilitated detection of CSA. This focus aligns with research which shows that appropriate responsiveness (Stiles et al. 1998), whereby the healthcare professional adjusts their practice to the unique situation and relationship with their clients, is an essential aspect of clinical competence. Therapists’ emphasis of time and opportunities to build a relationship with their clients to detect CSA are in line with young individuals’ own perspectives on disclosing CSA, whereas they describe a process of searching for a person to confide in that can be trusted, who will believe them, and who will be able to bear their stories (e.g., Alaggia, 2019; Brennan and McElvaney 2020; Lemaigre et al. 2017; Stige et al. 2022). These processes therefore take time. Standardised procedures, when used at an early stage in treatment, can therefore function as a barrier to disclosure, because the necessary relationship has not yet been established (Stige et al. 2022), or because the procedures themselves become an obstacle to establishing contact (Hagen et al. 2017).

Yet, standardised procedures are something that healthcare professionals are often required to follow, and the results showed that participants did this in all cases and were usually confident in doing so. Mandatory procedures may thus support healthcare professionals in their screening for CSA, as reflected in the fact that 31% of participants reported clearer procedures could enhance CSA detection. However, procedures alone were deemed insufficient to manage the complexity of CSA detection, and only 25% of the participants experienced that the current clinical practice at their workplace contributed to detection of CSA to a great extent. Satisfaction with current assessment practices was in turn significantly related to how often therapists assessed for CSA, as well as how often they reported to detect CSA. In addition, almost half of the participants identified fear of making mistakes or inappropriate questioning as significant barriers to CSA detection, while one-third viewed the fear of false CSA suspicion as a primary obstacle. Therefore, probing for CSA based on “only” intuition and clinical judgement may be too difficult for therapists as additional training in how to talk to young people about CSA, was considered the most crucial facilitator for CSA detection. These results therefore point to the inherent tension between procedures and clinical autonomy, and the significance of addressing this to find ways to improve CSA detection in CAP. How, then, can we build organisations that facilitate the detection of CSA? Can we develop support for healthcare professionals to improve their tolerance for the uncertainty and complexity of exploring CSA in ways that allow for responsiveness to the young person’s needs?

### Management level (CAP): how do we build organisations that facilitate detection of CSA?

As we have seen, therapists in this study required sustainable work environments that allowed for sufficient time to build a strong alliance with the child, and the autonomy to flexibly adjust their practice to each unique encounter to increase detection of CSA. This has direct implications for the organisation of services. In today’s mental healthcare system, CAP lies in a cross-pressure between multiple expectations and needs. CAP is expected to provide efficient and equal treatment to children and young people across a wide spectrum of disorders throughout the country and they are expected to provide cost-effective assessment and treatment to many children within a limited timeframe. This contrasts with research showing that uncovering CSA is a complicated and meticulous process, and that proceeding too quickly can be counterproductive (Alaggia et al. 2017; Flåm and Haugstvedt 2013; Stige et al. 2022). To best facilitate the uncovering of CSA, CAP managements must thus balance conflicting needs and allocate sufficient resources to potential CSA survivors.

However, the results also highlight the importance of organisations in providing sufficient support for healthcare professionals who work to uncover CSA. The study identified that improved knowledge on discussing CSA with children and adolescents, and better procedures were considered significant facilitators for detecting CSA. Additionally, therapists emphasised the importance of time and opportunity to discuss cases and share experiences with colleagues. This finding aligns with earlier research and shows that healthcare professionals may feel deeply isolated and alone when addressing potential CSA (Stige et al. 2022). Furthermore, it aligns with a tendency to rarely discuss emotionally demanding work in the healthcare system or the working conditions of healthcare personnel who work with such tasks (Breyer and Storms 2021). This underscores health care leaders’ responsibility to ameliorate these aspects and illustrates a potential pathway to increase detection of CSA in CAP.

Around one third of the therapists in this sample were uncertain about what to do if they detected CSA, with younger therapists both feeling more uncertain and experiencing less support. This has important implication for CAP. Research on therapist development (e.g., Rønnestad and Skovholt 2013; Rønnestad et al. 2018) shows that therapists develop through phases, and that each phase has its own challenges and developmental tasks. Early-career healthcare professionals are particularly vulnerable to balancing conflicting demands and expectations and handling complex cases like CSA independently. Professional reflection and learning from more experienced colleagues are therefore vital sources of development as a therapist (Rønnestad and Skovholt 2013). This points to possible paths to support younger therapists on an organisational level to facilitate continued therapist development and competent handling of complex cases, like CSA detection.

### The public services level: which agency is responsible for CSA detection?

Our results suggest a potential evasion of responsibility among various agencies involved with the child/youth. Participants noted that when more specialised agencies (e.g., CAP) were involved in a case, others often withdrew, relinquishing their responsibility. Conversely, several healthcare professionals believed that agencies other than CAP were better positioned to detect CSA. When engaged with a child/youth, the healthcare professionals felt responsible for uncovering CSA, and some found themselves left alone with this concern, even though they thought other agencies and professionals, e.g., teachers, who spend more time with the child, were better recipients for CSA disclosure. A major concern is the combination of a low CSA detection rate in CAP, other agencies that abdicate from CSA detection responsibility, and CAP professionals feeling inadequately supported and resourced to handle CSA assessment and detection.

In accordance with previous research, our findings point to a concerning lack of trust and cooperation between the agencies responsible for the child’s well-being in different areas. There was also uncertainty about CAP’s role in reporting CSA to LE, assessing recurrence risk and the urgency of reporting, informing caregivers, and establishing collaboration with the child/adolescent and parents about reporting abuse. Effective child protection requires a team approach, involving judicial, welfare, health and therapeutic interventions (Sedlak et al. 2006). A multi-disciplinary approach characterised by equal responsibility-sharing and reciprocal information exchange between agencies (e.g., LE, CPS, kindergarten/school, and CAP) (Nouman et al. 2020), could improve CSA detection and ensure comprehensive childcare. Each of these agencies makes distinctive and valuable contributions to CSA cases (Cleek et al. 2019).

### Strengths and limitations

In the present study we employed a mixed method design that enabled us to combine quantitative and qualitative data. By doing this, participants had the opportunity to add information and elaborate if response alternatives were missing. This combination of data strengthens the study’s validity. To further strengthen validity, we piloted the questionnaire by checking its comprehension and language among a relevant target population. Another strength of the study is our nationwide recruitment strategy, covering healthcare professionals’ experiences in all health regions in Norway. By recruiting in this way, however, we do not know exactly how many healthcare professionals received the survey, or what the precise response rate was. The sample size is small, which indicates a low response rate compared to the total number of potential CAP healthcare professionals in Norway. It is also a convenience sample, which may indicate that we reached those who wanted to express themselves, either because they were very committed to this work or because they had strong negative experiences. As such, the data may be biased towards therapists who are especially attentive to CSA. This may also limit the generalisability.

## Conclusions

Healthcare professionals in this study conveyed that uncovering CSA is deemed an important task in CAP, but that it is challenging, and that the procedures they currently have are not sufficient. There seems to be a tension between their use of standardised procedures and clinical judgement, reflected in therapists’ request for both clearer procedures and training, as well as more room for clinical judgement and more time to be able to establish a good alliance with the young person, to facilitate disclosure of CSA. The study highlights the complexity of uncovering CSA and emphasises some of the challenges that healthcare professionals, managements of CAP institutions and other public services must resolve, to increase the CSA detection rate.

### Clinical implications and directions for future research

Being mindful of the limitations of the study, it is nevertheless sensible to highlight some potential clinical implications. These will be presented as possible facilitators to increase the CSA detection rate in CAP. As discussed, these facilitators are primarily anchored at an organisational level and show that management must ensure therapists have good working conditions and can feel safe uncovering CSA. First, having sufficient time to establish a therapeutic relationship with the child/adolescent is fundamental to uncovering CSA within CAP. Time may strengthen healthcare professionals’ ability to create a safe space within which CSA survivors can disclose. Second, both standardised procedures and clinical judgement are useful tools for detection of CSA. Valuing both approaches as important is essential to facilitating CSA disclosure. Moreover, strengthening healthcare professionals’ clinical ability to identify and interpret more diffuse signs of CSA may increase early CSA detection. Third, time and arenas for discussion, reflection, and exchange of experience with colleagues are important, both for handling uncertainty and doubt, and for increasing competence. This may also augment healthcare personnel’s experience of support and community, which in turn can reduce their sense of being alone and their fear of making mistakes. This seems particularly important for younger and more inexperienced therapists. Fourth, it seems vital that therapists are given the opportunity to develop their clinical skills, both in terms of learning to talk to children/adolescents about CSA and increasing their trauma treatment skills. Finally, clarifying roles and responsibilities within the organisation and between agencies seems crucial. Interdisciplinary discussions and clearer guidelines on who does what, and when, and arenas for inter-agency cooperation, will hopefully improve the therapists’ ability to navigate the demanding process from assessing to detecting, and eventually reporting, CSA.

### Electronic supplementary material

Below is the link to the electronic supplementary material.

Supplementary Material 1

## Data Availability

The qualitative dataset generated and analyzed during the current study are not publicly available due to not compromising individual privacy and legal restrictions. The quantitative data are available from the corresponding author on reasonable request.

## Abbreviations

CAP: Child and Adolescent Psychiatry
CATS: Child and Adolescent Trauma Screen
CPS: Child Protective Services
CSA: Child Sexual Abuse
LE: Law Enforcement
