# Supplementary material for: Detecting child sexual abuse in child and adolescent psychiatry: a survey study of healthcare professionals’ assessment practice
Source: Int J Ment Health Syst. 2024 Apr 18;18:16. doi: 10.1186/s13033-024-00632-y (PMC11025146; doi:10.1186/s13033-024-00632-y)
Supplement: Supplementary file 1 — Supplementary Material 1 [file 13033_2024_632_MOESM1_ESM.docx]

**Supplementary material 1. Survey (translated)**

**Questionnaire (completion is regarded as consent to participation. Online survey):**

**Introduction:**

For several years now, there has been a focus on uncovering sexual abuse against children and young people, and employees in CAP must assess sexual abuse as part of the investigation. Little is known, however, about how CAP employees work to assess sexual abuse, and how they experience this work. This survey is a contribution to gaining more knowledge about this. The investigation is led by associate professor in clinical psychology at the University of Bergen, Signe Hjelen Stige. If you have any questions about the survey, contact the project manager Signe H. Stige on [Signe.Stige@uib.no](http://mailto/) 

Thank you very much for your help!

**Questions:**

0. For you to be able to withdraw consent and request access to, change and delete data, we ask you to create a code that only you know, which consists of the second letter of your mother's name, the month your father was born, last letter in your name and the third number in your mobile number: (open)

1. Gender (male/female/other)

2. Age (open)

3. Education (psychologist/doctor/social worker/educator/nurse/ family therapist/other)

4. When did you finish your education? (open)

5. Do you have a clinical specialty? (Yes/No)

5b. When did you finish your specialization? (open)

6. Which healthcare region do you work in (South-East/West/Mid/North)

7. How long have you worked in CAP? (open)

8. What job percentage do you have in CAP as of today? (open)

9. How many client consultations do you have on average per week? (open)

10. How often do you assess exposure to sexual abuse? (1-5; less often than once a month/ monthly/ weekly/ several times a week/ every day)

Elaborate (open)

11. What determines whether you start assessing possible sexual abuse? (multiple answers: procedure - everyone is asked / indication - observations or something the child says / others convey concern / clinical judgment - gut feeling or intuition / other)

12. What is most decisive for whether you start assessing possible sexual abuse? (procedure – everyone is asked/ indication – observations or something the child says/ others convey concern / clinical judgment – ​​gut feeling or intuition/ other)

13. What does it take for you to again assess/ask about sexual abuse where this has all been done at an earlier point in the treatment contact? (statements from the patient, observations, new information, input from colleagues/co-therapist, gut feeling or intuition / other)

14. How often does this happen? (1-5; less often than once a year/ a couple of times a year/ monthly/ several times a month/ weekly)

Elaborate on what determines whether assessing starts (open)

15. How do you usually detect sexual abuse? (multiple answers: using a form (e.g., CATS)/through conversation/through play, drawing etc./other)

16. What do you find most helpful when assessing sexual abuse? (multiple answers: scores on standardized forms (e.g., CATS)/conversations with children and parents/play, drawing etc./other)

17. To what extent do you know what to do if you suspect sexual abuse? (1-5; very often unsure/often unsure/occasionally unsure/mostly know what to do/know what to do)

Elaborate on how and what is most helpful (open)

18. How often do you discover sexual abuse in your clinical work / are you the first to learn that sexual abuse has occurred? (1-5; less often than once a year/ a couple of times a year/ monthly/ several times a month/ weekly)

19. What typically happens during treatment when it emerges that the child/young person has been exposed to sexual abuse? (Multiple choice: I report concerns to child protection / I am actively involved in reporting to the police / We take a break in the treatment while it is clarified what roles the various agencies will have / I continue the treatment without going directly into trauma symptoms and trauma history / I adjust the treatment focus and works directly with trauma symptoms and trauma history/other)

20. To what extent do you know what to do if you detect/find out about sexual abuse? (1-5; very often unsure/often unsure/occasionally unsure/mostly know what to do/know what to do)

Elaborate on experiences in discovering (open)

21. How often is assessing and uncovering sexual abuse a topic that is discussed in your workplace? (1-5; less often than once a year/ a couple of times a year/monthly/several times a month/weekly)

Elaborate (open)

22. To what extent do you feel that you have the support you need in the workplace when you work with assessing and uncovering sexual abuse? (1-5; to a very small extent/to a small extent/neither or/to a fairly large extent/to a very large extent)

Elaborate (open)

23. To what extent do you feel that current practices at your workplace contribute to discovering potential sexual abuse? (1-5; to a very small extent/to a small extent/neither or/to a fairly large extent/to a very large extent)

Elaborate (open)

24. From your perspective: What would have contributed to more sexual abuse being discovered? (multiple answers: clearer procedures/professional meeting point where suspicion can be discussed/colleague support/support from management/more professional knowledge/training or training in talking to children about CSA/more time or resources/better cooperation with other agencies (e.g., child protection, school, health center)/better cooperation with the police/other)

Elaborate (open)

25. From your perspective: To what extent does CAP have an important role in uncovering sexual abuse against children and young people? (1-5; to a very small extent/to a small extent/neither or/to some extent/to a large extent)

Elaborate (open)

26. From your perspective: What are the most important obstacles to being able to detect sexual abuse while the child or young person is in contact with CAP? (multiple answers: missing procedures/time pressure/discomfort connected to talking about sexual abuse/uncertainty about how to do things/lack of knowledge/afraid of making mistakes or asking questions in the wrong way/uncertain whether the child will feel better if they tell / afraid of falsely suspecting sexual abuse/ afraid of contributing to false memories/ other)

27. From your perspective: What is the biggest obstacle to discovering more sexual abuse within the CAP system? (lack of procedures/time pressure/discomfort linked to talking about sexual abuse/uncertainty about how to do things/lack of knowledge/fear of making mistakes or asking questions in the wrong way/unsure whether the child will feel better if they tell/fear for falsely suspecting sexual abuse/afraid of contributing to false memories/other)

Elaborate (open)

28. Are there other things you would like to share about assessing or uncovering sexual abuse in a CAP context? (open)

Thank you!

If you would like to know when the results will be published, send an email to [Signe.Stige@uib.no](http://mailto/)
